# Comprehensive bioinformatic analysis of HTR7: A potential biomarker for diagnosis, survival, and immunotherapy in pan-cancer

**DOI:** 10.1371/journal.pone.0335398

**Published:** 2025-11-14

**Authors:** Yuhao Yao, Xiao Xia, Lanxin Zhang, Hongtai Xiong, Size Li, Wei Hou

**Affiliations:** 1 Graduate School, Beijing University of Chinese Medicine, Beijing, China; 2 Department of Oncology, Guang’anmen Hospital, China Academy of Chinese Medical Sciences, Beijing, China; 3 Department of Cardiology, Guang’anmen Hospital, China Academy of Chinese Medical Sciences, Beijing, China; 4 Department of Gastroenterology, Dongzhimen Hospital, Beijing University of Chinese Medicine, Beijing, China; Sichuan University, CHINA

## Abstract

We used several public databases to perform a comprehensive pan-cancer analysis to determine the potential role of HTR7 in diagnosing tumors, predicting prognosis, and predicting cancer immunotherapy response. The results showed that HTR7 is highly expressed in 12 tumors and lowly expressed in 13 tumors compared with normal tissues. HTR7 has a specific diagnostic value in 18 cancers, especially in COAD, HNSC, KIRC, PCPG, and READ. High expression of HTR7 was associated with a favorable prognosis in ACC, COAD, KIRC, KIRP, PRAD, READ, SKCM, and THCA, while in CESC, ESCA, GBM, HNSC, PAAD, and THYM, high expression of HTR7 was associated with an unfavorable prognosis. In most tumors, HTR7 expression was positively correlated with the infiltration of monocytes, macrophages, and myeloid dendritic cells and negatively correlated with Th1 infiltration. We found that HTR7 expression was positively correlated with CD274, CTLA-4, HAVCR2, PDCD1, PDCD1LG2, and TIGIT in numerous tumors. Furthermore, our study showed that aberrant methylation of HTR7 was associated with the infiltration of many immune cells, including Th1, Th17, DC, macrophages, etc. In cancer pathways, HTR7 could inhibit the cell cycle, DNA damage, and hormone AR pathways and activate the EMT and RAS/MAPK pathways. GO and KEGG enrichment analyses revealed that HTR7 could participate in the G protein-coupled receptor signaling pathway, serotonin receptor signaling pathway, hormone signaling, cAMP signaling pathway, etc. Several drugs, including 5-fluorouracil, gemcitabine, sunitinib, tipifarnib, and trametinib, may be sensitive to high HTR7 expression in tumors.

## Introduction

Cancer has now become the leading cause of death and a significant barrier to longer life expectancy in humans [1]. The global burden of cancer incidence and mortality is increasing rapidly [2]. Exploring more precise and individualized treatments is a growing focus in cancer treatment today. The rise of cancer genomics and tumor microenvironment (TME) research has provided new ways of researching cancer [3,4], giving more cancers access to targeted therapy and immunotherapy.

Serotonin (5-hydroxytryptamine, 5-HT) is a biogenic monoamine whose role as a neurotransmitter in the central nervous system and as a motor mediator in the gastrointestinal tract is well established [5]. Many studies have shown that 5-HT has a potential stimulatory effect on tumor cell proliferation, invasion, dissemination, and tumor angiogenesis [6]. In addition, 5-HT has an immunomodulatory role in tumor immunity and effectively promotes signaling in immune cells and TME [6]. However, 5-HT has 14 isoforms, and there is a lack of research on the role of individual isoforms in the tumor area.

HTR7 mainly encodes the 5-HT7 receptor, and several studies have now shown that the 5-HT7 receptor is closely related to the development and progression of certain cancers, such as breast cancer, prostate cancer, non-small cell lung cancer, and laryngeal cancer [7–10]. Some drugs targeting the 5-HT7 receptor have shown preliminary evidence of inhibiting tumor growth and metastasis [8,11,12].

However, previous studies on HTR7 have only been conducted in a few specific cancers. There is a lack of comprehensive and in-depth pan-cancer analyses on HTR7, which is important for a full understanding of the role of HTR7 in cancers. Therefore, our study systematically analyzed the association of HTR7 with transcript levels, diagnostic value, clinicopathologic features, survival prognosis, TME, DNA methylation, cancer pathways, and potential drugs in 33 cancers, hoping to inform future research targeting HTR7.

## Materials and methods

### Gene expression analysis of HTR7

The TIMER2.0 database (http://timer.cistrome.org/) is a comprehensive resource for systematically analyzing immune infiltrates across diverse cancer types. It enables users to investigate the differential expression of any gene between tumor and adjacent normal tissues. Additionally, to compensate for the Cancer Genome Atlas (TCGA) data, we acquired RNA-seq data for 33 cancers from the TCGA database and Genotype-Tissue Expression (GTEx) database. The expression of HTR7 was evaluated using the downloaded data, and expression levels were compared between cancer samples and matched standard samples in 33 cancers. Expression data were Log2 transformed, and the Wilcoxon test was used to analyze the significant difference in expression; *P* < 0.05 indicated differential expression between tumor and normal tissues. Box plots were created using the R-packages “ggpubr” and “ggplot2”.

### Immunohistochemistry (IHC) and Immunofluorescence (IF) staining of HTR7

To evaluate differences in HTR7 expression at the protein level, IHC images of HTR7 protein expression in normal tissues and tumors tissues were downloaded from the Human Protein Atlas (HPA) database (https://www.proteinatlas.org/) and analyzed. Meanwhile, the subcellular location of HTR7 was explored through indirect IF microscopy from the HPA.

### Analysis of the relationships between HTR7 and diagnostic value and clinicopathologic features

Clinical data were downloaded from the TCGA. The receiver operating characteristic (ROC) curve was used to estimate the diagnostic value of HTR7 in pan-cancer using the R-package “pROC”. The closer the area under the curve (AUC) is to 1, the better the diagnostic accuracy is. AUC in 0.8–1 was considered highly diagnostic, and AUC in 0.6–0.8 was considered potentially diagnostic. The Wilcoxon test was used to investigate the correlation between HTR7 expression and clinicopathologic features, including clinical stage and histologic grade; *P* < 0.05 was considered statistically significant. Violin plots were drawn using the R-packages “ggpubr” and “ggplot2”.

### Analysis of the relationship between HTR7 expression and survival prognosis

Survival data were extracted for each sample downloaded from TCGA. We selected overall survival (OS), disease-specific survival (DSS), progression-free interval (PFI), and disease-free interval (DFI) to analyze the correlation between HTR7 expression and the cancer’s prognosis. Median OS time was calculated. The Kaplan-Meier method and Cox regression were used for survival analyses (*P* < 0.05) of each cancer type. Survival curves were generated using the R-packages “survival” and “survminer”.

### Analysis of the relationship between HTR7 expression and immunity

We downloaded STAR-counts data and corresponding clinical information from the TCGA (https://portal.gdc.cancer.gov). We then extracted data in TPM format and performed normalization using the log2(TPM + 1) transformation for further analysis. We used deconvolution to estimate immune cell concentrations and conduct precise immunological assessments [13]. We then acquired heat maps of Spearman’s correlations between HTR7 and different types of immune cells (*P* < 0.05 as significant). To investigate the impact of TME in tumors, immune and stromal scores were calculated to predict the correlation between HTR7 expression and stromal cells and immune cells. Moreover, we extracted the expression values of immune checkpoint genes to observe the expression patterns of HTR7 related to immune checkpoints. Spearman analysis correlated HTR7 expression and immune checkpoints (*P* < 0.05 as significant).

### DNA methylation analysis of HTR7

We searched the UALCAN database (http://ualcan.path.uab.edu/) to investigate HTR7 promoter DNA methylation levels in cancers and to determine the differences between tumors and normal tissues; *P* < 0.05 was considered statistically significant. Shiny Methylation Analysis Resource Tool (SMART, http://www.bioinfo-zs.com/smartapp/), an interactive web server for analyzing DNA methylation of the TCGA project, was applied to discuss the distribution of methylation [14]. Using survival curves, we also analyzed the correlation between DNA methylation levels and survival. Furthermore, we estimated the association between gene methylation and immune cell infiltrates using Spearman analysis (*P* < 0.05 was considered significant).

### Analysis of the relationship between HTR7 and cancer pathways activity

The Gene Set Variation Analysis (GSVA) score represents the integrated level of gene set expression, which is positively correlated with the expression of the gene set [15]. We explored the correlation between the GSVA score calculated using the R-package “GSVA” and the activity of well-known cancer-related pathways, including TSC/mTOR, RTK, RAS/MAPK, PI3K/AKT, hormone ER, hormone AR, EMT, DNA damage response, cell cycle, and apoptosis pathways. Reverse phase protein array (RPPA) is a high-throughput antibody-based technique with procedures similar to Western blots [16]. Proteins are extracted from tumor tissue or cultured cells, denatured with SDS, and printed onto nitrocellulose-coated slides, followed by the application of an antibody probe. RPPA data from the TCPA database (https://www.tcpaportal.org/tcpa/) were used to calculate the pathway activity scores of 10 cancer-related pathways for 7,876 samples (from the TCGA database) across 32 cancer types.

### Co-expressed genes and enrichment analysis of HTR7

GeneMANIA (http://genemania.org/) is an online tool for exploring gene interactions and functions and identifying co-expressed genes [17]. Twenty genes co-expressed with HTR7 were obtained through GeneMANIA. Gene Ontology (GO) and Kyoto Encyclopedia of Genes and Genomes (KEGG) enrichment analyses for HTR7-correlated genes were conducted using the R-package “Cluster Profile”.

### Genetic alteration analysis of HTR7

cBioPortal (https://www.cbioportal.org/) is a web platform that provides a resource for exploring, visualizing, and analyzing multidimensional cancer genomics data [18]. We performed the pan-cancer genetic alteration analysis of HTR7 using this database. The genetic alterations and mutation site information were explored with the “Oncoprint”, “Cancer Type Summary,” and “Mutations” modules.

### Drug sensitivity analysis

GSCALite (https://guolab.wchscu.cn/GSCA/#/) is an integrated platform for genomic, pharmacogenomic, and immunogenomic gene set cancer analysis [19]. Using Pearson correlation analysis, we utilised this data to determine the correlation between gene mRNA expression and the drug’s 50% inhibitory concentration (IC50). IC50 is the drug concentration required to inhibit a specific biological activity by 50% in vitro. It measures drug potency—the lower the IC50, the more potent the compound.

### Statistics analysis

The Wilcoxon test was applied to investigate HTR7 expression and its correlation with clinical characteristics according to the TCGA and GTEx databases; *P* < 0.05 indicated the statistical significance. This study used the Kaplan-Meier curve, log-rank test, and Cox regression model for all survival analyses. The correlation analysis between the two variables used Spearman’s or Pearson’s test; *P* < 0.05 was considered significant. All statistical analyses were processed by the R software (Version 4.5.0).

## Results

### Differential expression of HTR7 between tumor and normal tissue samples

**Fig 1:**
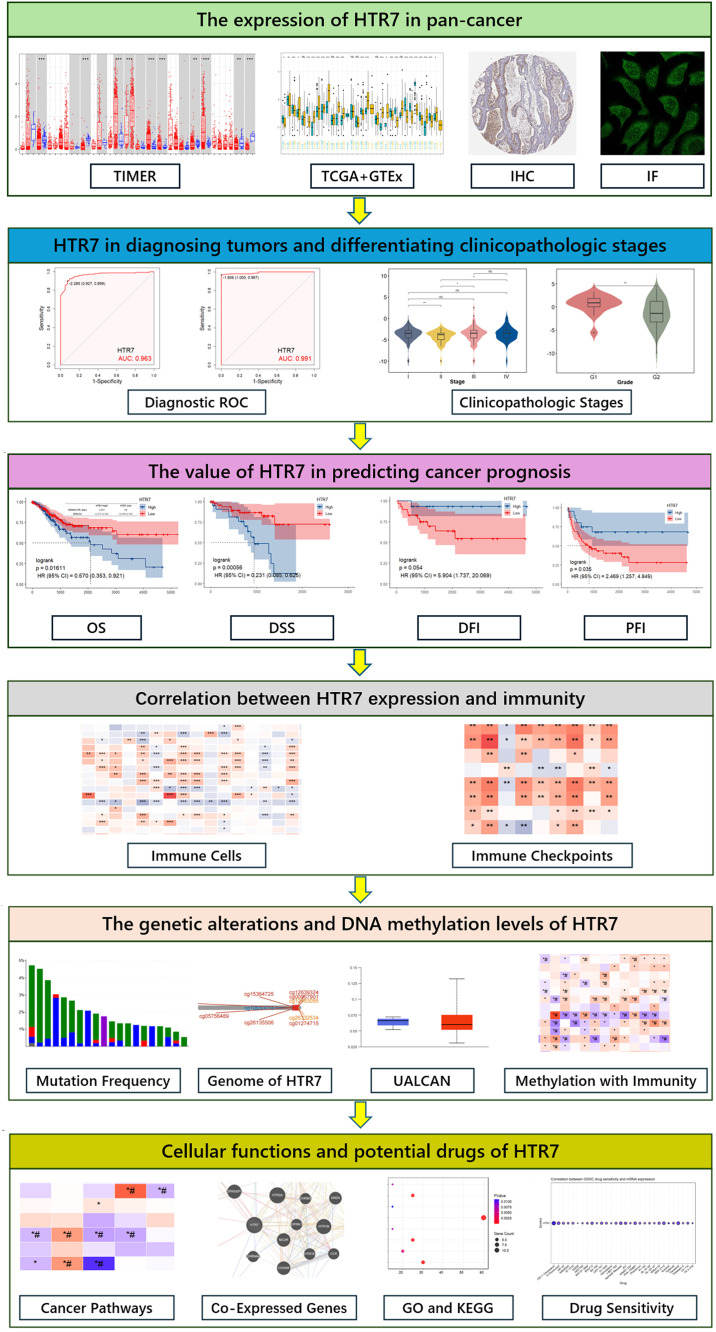
The workflow of this study.

**Fig 2:**
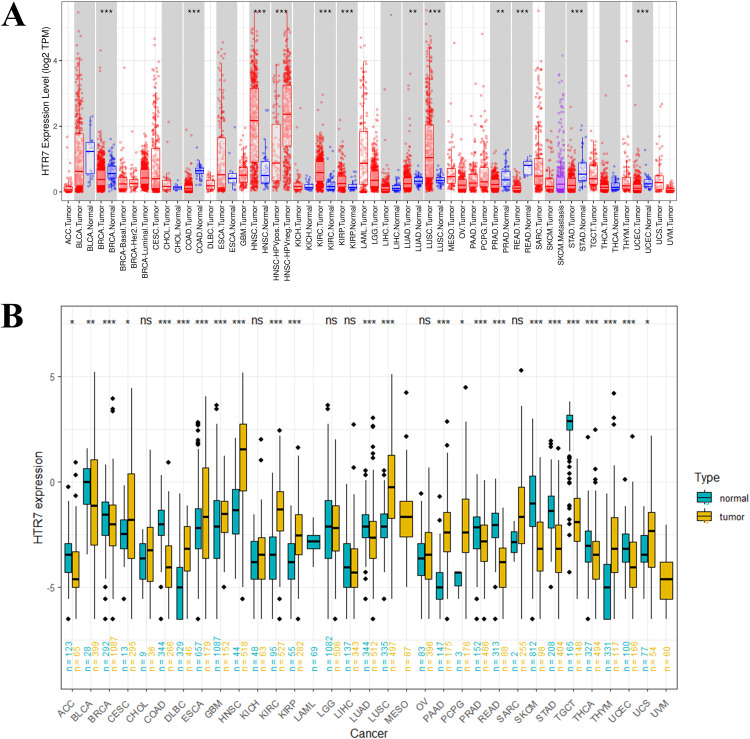
HTR7 expression in pan-cancer from different databases. **(A)** HTR7 expression in pan-cancer in TIMER. HTR7 expression was downregulated in BRCA, COAD, LUAD, PRAD, READ, STAD, and UCEC, and upregulated in HNSC, KIRC, KIRP, and LUSC. **(B)** HTR7 expression in TCGA + GTEx. HTR7 expression was downregulated in ACC, BLCA, BRCA, COAD, LUAD, PRAD, READ, STAD, SKCM, TGCT, THCA, and UCEC, and upregulated in CESC, DLBC, ESCA, GBM, HNSC, KIRC, KIRP, PAAD, PCPG, LUSC, THYM, and UCS. The Wilcoxon test was used. (*, *P* < 0.05; **, *P* < 0.01; ***, *P* < 0.001, ns: no statistical. TCGA, the Cancer Genome Atlas; GTEx, Genotype-Tissue Expression).

The flowchart of our study is shown in Fig 1. To explore the expression level of HTR7 in pan-cancer, we used the TIMER and TCGA + GTEx databases. In the TIMER database, HTR7 expression was downregulated in BRCA, COAD, LUAD, PRAD, READ, STAD, and UCEC, and upregulated in HNSC, KIRC, KIRP, and LUSC (Fig 2A). To compensate for the inadequacy of the TIMER database data, we combined TCGA with GTEx databases, and the results showed that HTR7 expression was downregulated in ACC, BLCA, BRCA, COAD, LUAD, PRAD, READ, SKCM, STAD, TGCT, THCA, UCEC, and upregulated in CESC, DLBC, ESCA, GBM, HNSC, KIRC, KIRP, PAAD, PCPG, LUSC, THYM, UCS (Fig 2B). The results of the TIMER and TCGA+GTEx databases showed high consistency.

### IHC and IF staining of HTR7

**Fig 3:**
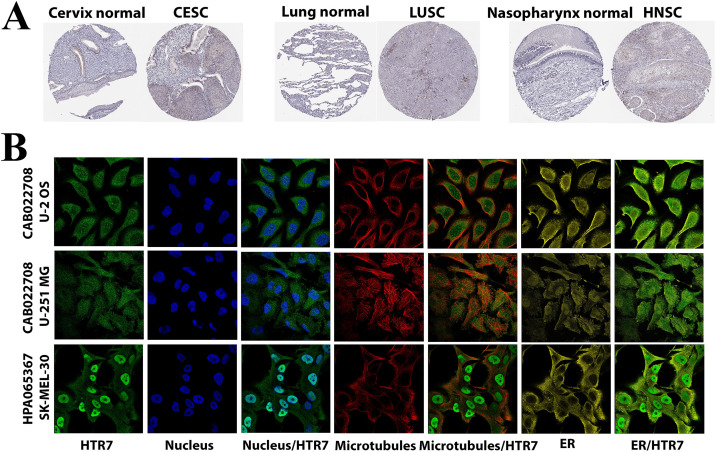
IHC and IF staining of HTR7. **(A)** The different expressions of HTR7 between normal tissues and tumor tissues in CESC, LUSC, and HNSC. **(B)** IF staining of the subcellular localization of HTR7. HTR7 was localized to the plasma membrane, nucleoplasm, nuclear speckles, cytosol, and primary cilium.

To explore the protein expression level of HTR7, we downloaded IHC and IF images from the HPA dataset. The results showed that HTR7 was mainly enriched in HNSC, CESC, LUSC, and ESCA (Fig 3A). IF obtained the subcellular localization of HTR7. HTR7 was mainly localized to the plasma membrane. In addition, it was localized to the nucleoplasm, nuclear speckles, cytosol, and primary cilium (Fig 3B).

### High diagnostic value of HTR7 in pan-cancer

**Fig 4:**
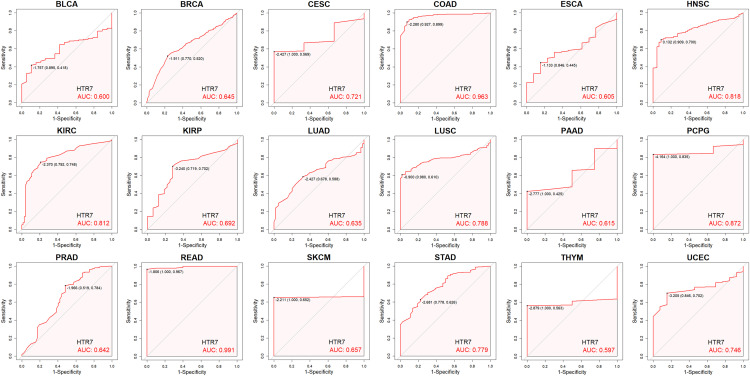
Diagnostic ROC curve of HTR7 in tumor diagnosis. HTR7 had high diagnostic value in COAD, HNSC, KIRC, PCPG, and READ (AUC > 0.8), and potential value in 13 tumors (0.6 < AUC < 0.8). ROC, receiver operating characteristic; AUC, the area under the curve.

We used the diagnostic ROC curve to explore the specificity and sensitivity of HTR7 in diagnosing tumors. The results showed that HTR7 had certain diagnostic accuracy (AUC > 0.6) in 18 cancer types. Among them, HTR7 had high diagnostic values in COAD, HNSC, KIRC, PCPG, and READ (AUC > 0.8) and potential values in BLCA, BRCA, CESC, ESCA, KIRP, LUAD, LUSC, PAAD, PRAD, SKCM, STAD, THYM, and UCEC (0.6 < AUC < 0.8) (Fig 4). Especially in COAD and READ, the AUC reached 0.963 and 0.991.

### HTR7 expression varies in clinicopathologic staging

**Fig 5:**
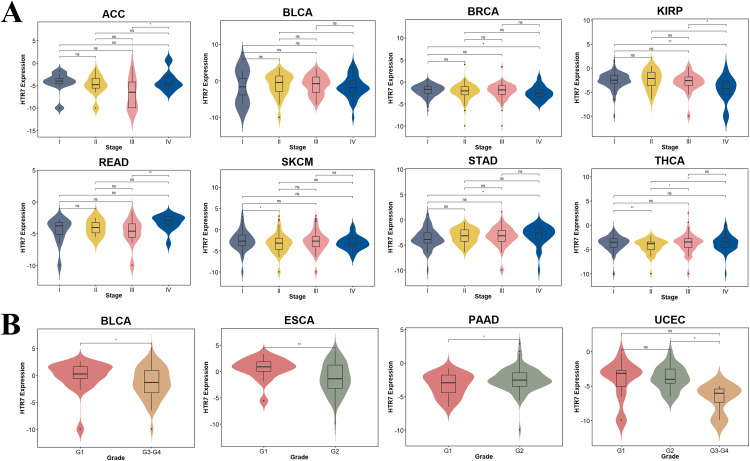
The relationship between HTR7 expression and clinicopathologic staging. **(A)** Differences in HTR7 expression were observed in the clinical stages of different tumors. **(B)** Differences in HTR7 expression were found in the pathological stages of a few tumors. The Wilcoxon test was used. (*, *P* < 0.05; **, *P* < 0.01, ns: no statistical).

We then explored the correlation of HTR7 expression with clinical staging and pathological grading. Regarding clinical staging, we found that high HTR7 expression was significantly associated with clinical stage IV in ACC, READ, and STAD. However, in clinical stage IV in BLCA, BRCA, and KIRP, HTR7 was lowly expressed. In SKCM, however, high HTR7 expression was closely associated with clinical stage I. In THCA, HTR7 was highly expressed in both clinical stages I and III to IV (Fig 5A). Regarding pathological grading, in BLCA and ESCA, low-grading (G1) tumors highly expressed HTR7. In PAAD, intermediate grading (G2) tumors highly expressed HTR7. In UCEC, high-grading (G3-G4) tumors had significantly lower HTR7 expression (Fig 5B).

### High prognostic value of HTR7 in pan-cancer

**Fig 6:**
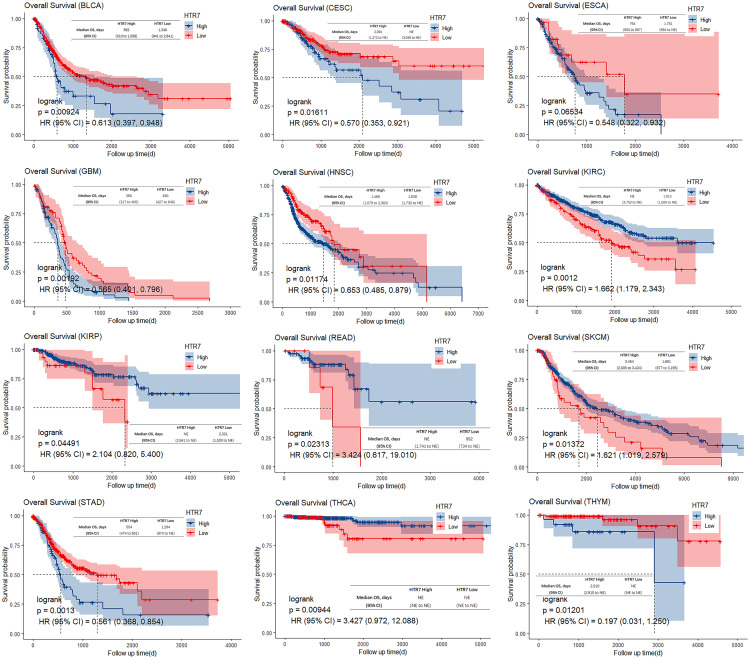
The univariate regression and Kaplan−Meier curves for OS in pan-cancer. High HTR7 expression was associated with unfavorable OS in BLCA, CESC, ESCA, GBM, HNSC, STAD, and THYM. High HTR7 expression was associated with favorable OS in KIRC, KIRP, READ, SKCM, and THCA. The Kaplan-Meier method and Cox regression were used.

To study the association between HTR7 expression level and prognosis, we performed a survival association analysis for each cancer, including OS, DSS, PFI, and DFI. In terms of OS, high HTR7 expression was associated with unfavorable OS in BLCA, CESC, ESCA, GBM, HNSC, STAD, and THYM, and conversely, high HTR7 expression was associated with favorable OS in KIRC, KIRP, READ, SKCM, and THCA (Fig 6). In terms of DSS, high HTR7 expression was associated with unfavorable DSS in BLCA, ESCA, GBM, HNSC, PAAD, STAD, THYM, and UVM, and conversely, high HTR7 expression was associated with favorable DSS in ACC, KIRC, KIRP, LUSC, PRAD, READ, and SKCM (S1 Fig). In terms of PFI, high HTR7 expression was associated with unfavorable PFI in BLCA, BRCA, GBM, HNSC, PAAD, STAD, THYM, and UVM, and conversely, high HTR7 expression was associated with favorable PFI in ACC, CESC, COAD, KIRC, KIRP, and UCS (S2 Fig). In terms of DFI, high HTR7 expression was associated with unfavorable DFI in BLCA, DLBC, and ESCA. In contrast, conversely, high HTR7 expression was associated with favorable DFI in ACC, CESC, COAD, LUSC, PRAD, TGCT, UCS, and UVM (S3 Fig).

These results suggest that HTR7 expression is highly valuable in predicting the prognosis of ACC, BLCA, CESC, ESCA, GBM, HNSC, KIRC, KIRP, STAD, THYM, and UVM.

### Strong association of HTR7 with immune cell infiltration and immune checkpoint inhibitor

**Fig 7:**
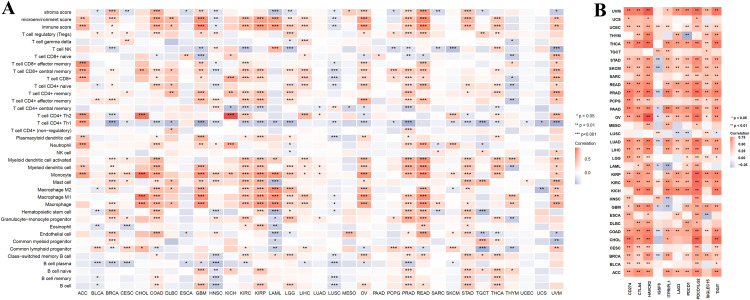
Correlation analysis between HTR7 and immunity in pan-cancer. **(A)** Correlation between HTR7 and immune cells. HTR7 expression was positively correlated with monocyte, macrophage, and myeloid dendritic cell infiltration and negatively correlated with Th1 infiltration. **(B)** Correlation between HTR7 and immune checkpoints. HTR7 expression was positively correlated with CD274, CTLA-4, HAVCR2, PDCD1, PDCD1LG2, and TIGIT in most tumors. Spearman analysis was used. (*, *P* < 0.05; **, *P* < 0.01; ***, *P* < 0.001).

Considering the critical role of immune infiltration in oncology progression, we explored the association of HTR7 expression with immune cell infiltration and immune checkpoints in pan-cancer. In 21 cancers, HTR7 and monocyte infiltration were found to be positively correlated. In 17 cancers, HTR7 and macrophage infiltration were found to be positively correlated. Regarding macrophage subtypes, HTR7 expression was positively correlated with M1 infiltration alone in CHOL, LIHC, PRAD, and THYM, while it was positively correlated with M2 infiltration alone in BRCA, DLBC, and STAD. In 16 cancers, HTR7 and myeloid dendritic cell infiltration were found to be positively correlated. In 21 cancers, HTR7 and Th1 infiltration were negatively correlated (Fig 7A).

Regarding individual cancers, in PRAD, HTR7 was associated with the infiltration of 27 immune cells. In COAD and KIRC, HTR7 was associated with infiltrating 22 immune cells. In UVM and READ, HTR7 was associated with the infiltration of 21 immune cells. In GBM, HTR7 was associated with the infiltration of 20 immune cells. In BRCA, THCA, and HNSC, HTR7 was associated with infiltrating 19 immune cells. We calculated the stromal score and immune score for 33 cancers. The immune score was higher in ACC, BRCA, COAD, DLBC, GBM, KIRC, KIRP, LAML, LGG, LIHC, OV, PRAD, READ, STAD, THCA, and UVM. In HNSC and LUSC, the immune score was lower. The stromal score was higher in BRCA, COAD, GBM, MESO, OV, PRAD, READ, and STAD, while lower in CESC, ESCA, HNSC, LAML, LGG, LUSC, PCPG, SARC, TGCT, and UVM (Fig 7A).

Regarding immune checkpoints, we found that HTR7 expression was positively correlated with CD274, CTLA-4, HAVCR2, PDCD1, PDCD1LG2, and TIGIT in most tumors (Fig 7B). In ACC, BRCA, COAD, KIRC, KIRP, LIHC, LUAD, OV, PAAD, PRAD, READ, SKCM, STAD, THCA, UCEC, UVM, HTR7 expression was positively correlated with at least eight immune checkpoints. While in DLBC, ESCA, HNSC, LUSC, MESO, TGCT, THYM, and UCS, HTR7 expression was associated with fewer immune checkpoints (Fig 7B).

The above results suggest that ACC, BRCA, COAD, KIRC, KIRP, LIHC, OV, PRAD, READ, STAD, THCA, and UVM may have immunotherapeutic potential. In contrast, HNSC and LUSC may have poor immunotherapeutic effects.

### Correlation of DNA methylation of HTR7 with tumor prognosis and immune cell infiltration

**Fig 8:**
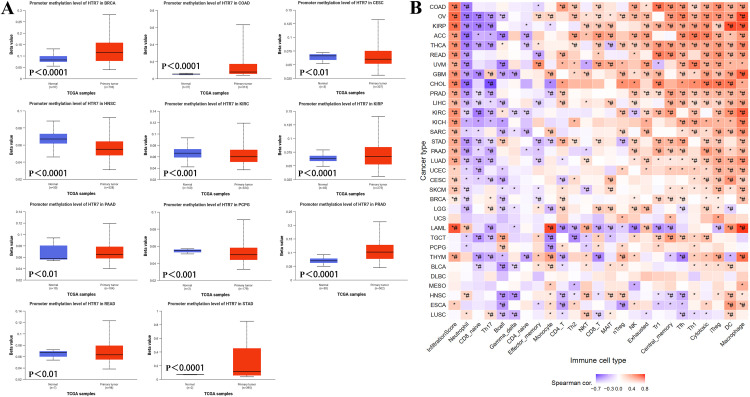
Correlation of DNA methylation with HTR7 and immune cell infiltration. **(A)** The promoter methylation level of HTR7 in cancers. The promoter methylation level was significantly lower in CESC, HNSC, KIRC, and PCPG. The promoter methylation level was increased in BRCA, COAD, PRAD, and STAD. **(B)** The association of HTR7 methylation with immune cell infiltration. HTR7 methylation was positively correlated with Th1, cytotoxic, iTreg, DC, and macrophage infiltration and negatively correlated with neutrophil, CD8, Th17, and B cell infiltration. Spearman analysis was used. (*, *P* < 0.05).

DNA methylation has been shown to play a crucial role in the development and progression of cancers. We first explored the promoter methylation level of HTR7 in cancers. The results showed that promoter methylation levels were significantly lower in CESC, HNSC, KIRC, and PCPG compared with normal tissues, which may have led to the high expression of HTR7 in these tumors. In BRCA, COAD, PRAD, and STAD, the promoter methylation level was increased, which may have led to the decreased expression of HTR7 (Fig 8A).

HTR7 had 11 methylation probes, including cg10650018, cg05756489, cg06291867, cg15364725, cg01274715, cg00967901, cg26135506, cg26332534, cg12583095, cg23720528 and cg12639324 (S4 Fig). We explored the correlation between the expression of these 11 methylated probes and cancer prognosis (Table 1). cg10650018 Hypermethylation is associated with a worse prognosis in ACC and SKCM. cg05756489 Hypermethylation is associated with a worse prognosis in ACC, KIRC, KIRP, PAAD, PRAD, and SKCM. cg06291867 Hypermethylation is associated with a worse prognosis in ACC, KIRC, KIRP, LUAD, PAAD, and THCA but a better prognosis in CESC. cg15364725 Hypermethylation is associated with a worse prognosis in BLCA, KIRP, and THCA but a better prognosis in STAD. cg01274715 Hypermethylation is associated with a worse prognosis in KIRC, UCEC, and UVM but a better prognosis in BRCA. cg00967901 Hypermethylation is associated with a worse CESC prognosis and a better KIRC prognosis. cg26135506 Hypermethylation is associated with better prognosis in GBM and KIRC. cg26332534 Hypermethylation is associated with a worse prognosis in KIRP and a better prognosis in STAD. cg12639324 Hypermethylation is associated with a better prognosis in STAD. cg12583095 Hypermethylation is associated with a worse prognosis for UVM. Overall, in most cancers, Hypermethylation of the methylation probes of HTR7 was associated with a worse prognosis.

**Table 1:** Relationship between HTR7 methylated CpG and survival.

Cancer	CpG	*HR (95% CI)*	*P*
ACC	cg10650018	2.75 (1.3 - 5.81)	0.0081
	cg05756489	2.64 (1.25–5.57)	0.0119
	cg06291867	2.8 (1.33–5.92)	0.007
BLCA	cg15364725	1.53 (1.14–2.05)	0.0046
BRCA	cg01274715	0.64 (0.44–0.95)	0.0218
CESC	cg06291867	0.6 (0.38–0.96)	0.0303
	cg00967901	1.72 (1.08–2.73)	0.0252
GBM	cg26135506	0.67 (0.46–0.99)	0.0419
KIRC	cg05756489	1.97 (1.34–2.89)	9e-04
	cg06291867	1.67 (1.13–2.45)	0.0112
	cg26135506	0.58 (0.39–0.85)	0.0049
	cg00967901	0.68 (0.46–1)	0.0447
	cg01274715	1.76 (1.2–2.59)	0.0051
KIRP	cg05756489	3.44 (1.82–6.52)	3e-04
	cg06291867	1.97 (1.04–3.73)	0.0391
	cg15364725	2.64 (1.4–4.99)	0.006
	cg26332534	2.3 (1.22–4.35)	0.0136
LUAD	cg06291867	1.46 (1.07–2)	0.0152
PAAD	cg05756489	1.71 (1.15–2.55)	0.0079
	cg06291867	1.67 (1.13–2.49)	0.0106
PRAD	cg05756489	6.92 (1.98–24.16)	0.0302
SKCM	cg10650018	1.44 (1.1 - 1.88)	0.0079
	cg05756489	1.61 (1.23–2.1)	6e-04
STAD	cg15364725	0.64 (0.46–0.88)	0.0062
	cg12639324	0.69 (0.5–0.96)	0.0253
	cg01274715	0.68 (0.49–0.94)	0.0197
	cg26332534	0.64 (0.47–0.89)	0.0077
THCA	cg06291867	8.75 (3.26–23.46)	5e-04
	cg15364725	3.75 (1.4–10.05)	0.0136
UCEC	cg01274715	1.7 (1.06–2.7)	0.0286
UVM	cg01274715	2.34 (1.03–5.32)	0.0441
	cg12583095	2.44 (1.07–5.55)	0.0341

Then, we analyzed the correlation between HTR7 gene methylation and the infiltration of 24 immune cells. In most tumors, HTR7 methylation was positively correlated with Th1, cytotoxic, iTreg, DC, and macrophage infiltration and negatively correlated with neutrophil, CD8, Th17, and B cell infiltration (Fig 8B). This result is similar to the level of HTR7 expression and immune cell infiltration.

Analyzing specific cancers, HTR7 methylation was associated with more immune cell infiltration in COAD, OV, KIRP, ACC, THCA, READ, UVM, and GBM. While in LUSC, ESCA, HNSC, THYM, and LAML, HTR7 methylation was negatively associated with immune cell infiltration (Fig 8B).

### Effects of HTR7 on different signaling pathways in pan-cancer

**Fig 9:**
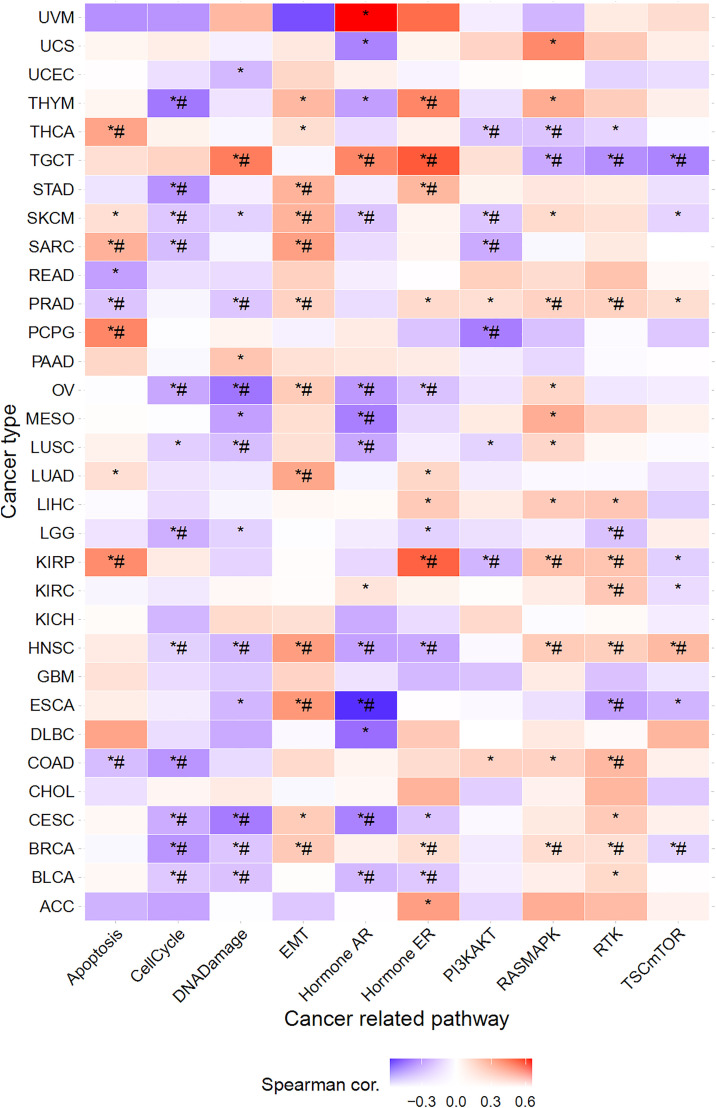
Correlation analysis between HTR7 and cancer pathways in pan-cancer. HTR7 could inhibit the cell cycle, DNA damage, and hormone AR pathways in most cancers and activate the EMT and RAS/MAPK pathways. (*, *P* < 0.05).

We explored the impact of HTR7 expression on the activity of several well-known cancer pathways. The results showed that HTR7 could inhibit the cell cycle, DNA damage, and hormone AR pathways in most cancers and activate the EMT and RAS/MAPK pathways. In some cancers, apoptosis, hormone ER, and RTK pathways were activated and inhibited in some cancers (Fig 9).

Regarding specific cancers, HTR7 expression activated or inhibited multiple BRCA, CESC, HNSC, KIRP, OV, PRAD, SKCM, and TGCT cancer pathways (Fig 9).

The above results reveal the influence of HTR7 gene expression on cellular function, which may provide a reference for targeted cancer therapy.

### Co-expressed genes and enrichment analysis of HTR7

To more comprehensively analyze the influence of HTR7 on cancers, we explored the genes related to HTR7 and performed GO and KEGG enrichment analyses. The results showed that 20 genes were closely related to HTR7, involving multiple networks, including physical interactions, co-expression, and co-localization (S5 Fig).

**Fig 10:**
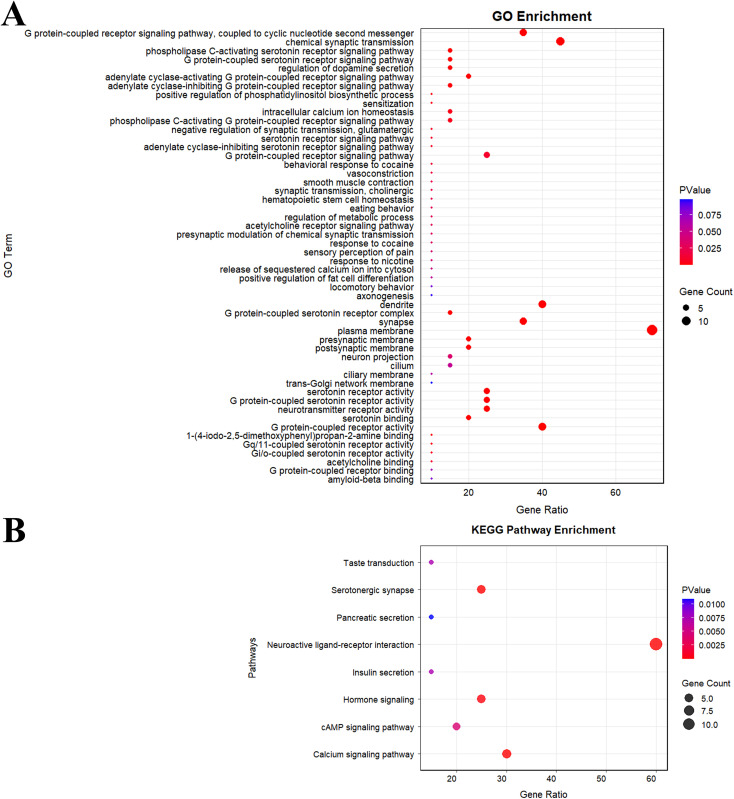
Enrichment analysis of HTR7-related genes. **(A)** GO (BP, CC, MF) enrichment analysis of HTR7-related genes. **(B)** KEGG enrichment analysis of HTR7-related genes.

Biological process (BP) enrichment analysis suggested that HTR7-related genes were involved in the chemical synaptic transmission, the G protein-coupled receptor signaling pathway, the serotonin receptor signaling pathway, the regulation of dopamine secretion, the intracellular calcium ion homeostasis and the adenylate cyclase-modulating G protein-coupled receptor signaling pathway (Fig 10A). Cellular component (CC) analysis showed that HTR7-related genes were involved in the plasma membrane, the dendrite, the synapse, the presynaptic membrane, the postsynaptic membrane, the neuron projection, and the G protein-coupled serotonin receptor complex (Fig 10A). In molecular function (MF) analysis, results showed that HTR7-related genes were mainly involved in neurotransmitter receptor activity, serotonin receptor activity, G protein-coupled serotonin receptor activity, G protein-coupled receptor activity, and serotonin binding (Fig 10A).

KEGG pathway analysis revealed that HTR7-related genes primarily participated in the neuroactive ligand-receptor interaction pathway, with a few genes also involved in other pathways, including the serotonergic synapse, calcium signaling pathway, hormone signaling, cAMP signaling pathway, taste transduction, pancreatic secretion, and insulin secretion. (Fig 10B).

### Genetic alteration analysis of HTR7

**Fig 11:**
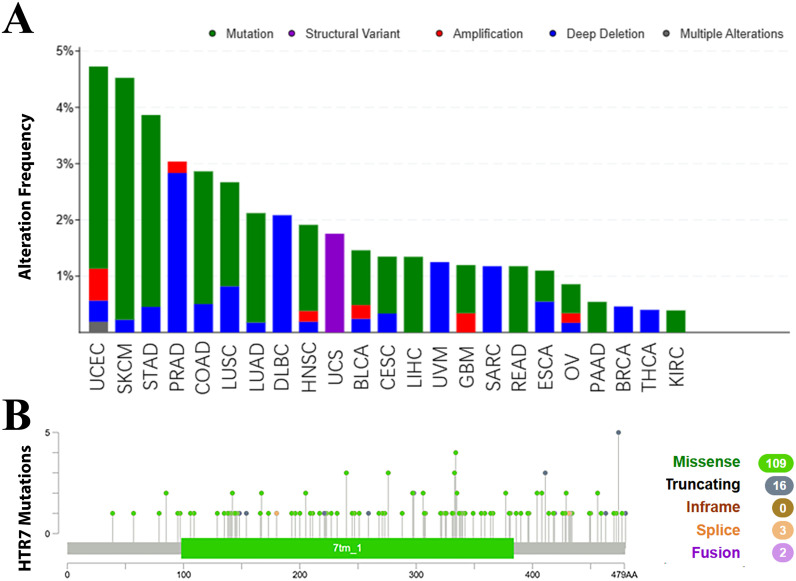
Genetic alterations of HTR7 in pan-cancer. **(A)** The frequency of HTR7 gene variants was highest in UCEC and SKCM. Mutations were most common in the vast majority of cancers. **(B)** Mutation types, number, and sites of HTR7 across protein domains.

To explore HTR7 gene variants in pan-cancer, we utilised the cBioPortal platform, which revealed that the frequency of HTR7 gene variants was highest in UCEC and SKCM (＞4%). Mutations were most common in the vast majority of cancers; deep deletions predominated in PRAD, DLBC, UVM, SARC, BRCA, and THCA. In UCS, structural variants occurred predominantly (Fig 11A). In addition, we found 130 mutation sites with missense mutation as the primary alteration type in HTR7 (Fig 11B).

### Potential drugs targeting HTR7

**Fig 12:**
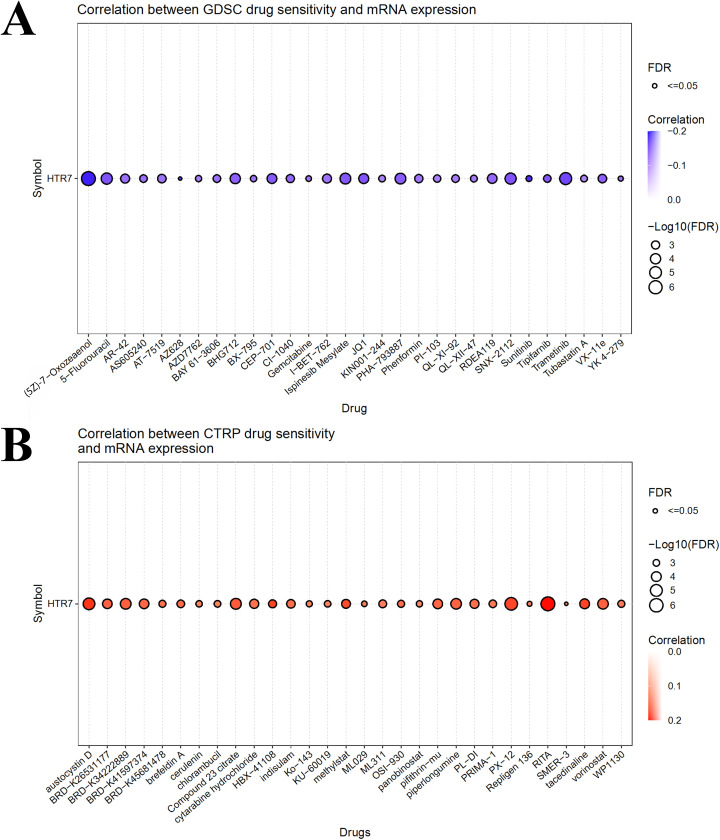
The associations of HTR7 expression and drug sensitivity. **(A)** 5-fluorouracil, gemcitabine, sunitinib, tipifarnib, and trametinib may be sensitive to high HTR7 expression, as indicated by GDSC. **(B)** Chlorambucil, hydrochloride, insular, and vorinostat may resist high HTR7 expression based on CTRP. IC50 is the drug concentration required to inhibit a specific biological activity by 50% in vitro. It measures drug potency—the lower the IC50, the more potent the compound.

We performed drug sensitivity analyses targeting HTR7 expression. The results from Genomics of Drug Sensitivity in Cancer (GDSC) showed that HTR7 expression was negatively correlated with IC50 of a larger number of drugs, including 5-fluorouracil, gemcitabine, sunitinib, tipifarnib, and trametinib, implying that these drugs may be sensitive to high HTR7 expression (Fig 12A). The data from the Cancer Therapeutics Response Portal (CTRP) showed that HTR7 expression was positively correlated with IC50 of several drugs, including chlorambucil, hydrochloride, insular, and vorinostat, implying that these drugs may be resistant to high HTR7 expression (Fig 12B).

## Discussion

HTR7, the gene encoding the 5-HT7 receptor, a G protein-coupled receptor, has been shown in several studies to be associated with cancer development. We comprehensively explored the correlation between HTR7 and 33 cancers, including gene expression, diagnostic value, prognosis, clinical and pathological staging, TME, DNA methylation, cancer pathways, and drug sensitivity, using databases including TCGA, GTEx, UALCAN, HPA, and cBioportal.

In terms of gene expression, the results of the TIMER and TCGA + GTEx databases showed high concordance. The discrepancies in HTR7 expression between the TIMER and GTEx + TCGA databases may stem from differences in sample sources, techniques, or sample preprocessing protocols. Combining these two analyses, HTR7 is highly expressed in 12 tumors and lowly expressed in 13 tumors. According to our knowledge, this study is the first to illustrate the dual oncogenic and tumor-suppressive potential of HTR7 across many cancer types, particularly uncovering previously unreported high expression in malignancies like KIRC, KIRP, PAAD, PCPG, and UCS. The dual function of HTR7 may be elucidated by its context-dependent interactions with signaling pathways, including the RAS/MAPK and AR/hormonal signaling pathways. Subsequent mechanistic investigations targeting these pathways could clarify these context-specific distinctions.

The differential expression of HTR7 in tumor tissues has prompted investigations into its diagnostic value for cancers. Our study suggests that HTR7 has a specific diagnostic value in 18 cancers, especially in COAD, HNSC, KIRC, PCPG, and READ. In terms of clinical staging, we found that the expression of HTR7 in BLCA, BRCA, and SKCM was negatively correlated with the clinical staging, and HTR7 was also lowly expressed in these tumor tissues, which further suggests that HTR7 may have a cancer-suppressive role in BLCA, BRCA, and SKCM. However, there are differences in the expression levels of HTR7 in different pathological types of BRCA. Dandan Zhan’s study demonstrated that HTR7 expression was inhibited in invasive mixed breast carcinoma [20], whereas Venhar Cınar’s study revealed that HTR7 expression was increased in triple-negative breast cancer (TNBC) [7]. Available studies suggest that HTR7 has dual genetic properties.

Subsequently, we further analyzed whether the expression levels of HTR7 influence cancer prognosis. It was found that high expression of HTR7 was associated with a favorable prognosis in ACC, COAD, KIRC, KIRP, PRAD, READ, SKCM, and THCA. It is noteworthy that HTR7 was lowly expressed in all of these tumors, which once again suggests that HTR7 may play a cancer-suppressive role in these tumors. A study by Harrison M Penrose et al. confirmed that in colon cancer, low HTR7 expression was associated with substantially poorer patient survival [21]. In CESC, ESCA, GBM, HNSC, PAAD, and THYM, high expression of HTR7 was associated with an unfavorable prognosis. Research by Xiaoli Sheng et al. confirmed that high HTR7 expression was associated with an unfavorable prognosis in laryngeal cancer [10]. Clinical research by Marta Rodríguez et al. [22] indicated that high levels of HTR7 mRNA in exosomal pools were associated with a poor prognosis in breast cancer patients, consistent with our findings (S2 Fig). Considering the continuous link between HTR7 expression and the prognosis of patients with various malignancies, developing a clinical-grade HTR7 expression test as a component of a comprehensive prognostic biomarker panel may be an important direction for future research.

TME and immunity are key factors that promote tumour growth and metastasis, which have been increasingly emphasized and studied. In the analysis of TME, our findings showed that in most cancers, HTR7 expression was positively correlated with infiltration of monocytes, macrophages, and myeloid dendritic cells and negatively correlated with Th1 infiltration. Monocyte and macrophage infiltration have been reported to be closely associated with cancer development and progression [23]. Monocytes can adapt to tumor-derived stimuli, likely driving their pro-tumorigenic properties [23,24]. Monocytes and monocyte-derived cells can promote tumor growth and metastasis by inducing immune tolerance, promoting angiogenesis, remodeling the extracellular matrix (ECM), and inducing epithelial-mesenchymal transition (EMT) [25]. Tumor-associated macrophages (TAMs) are an important component of the tumor microenvironment and play an important role in tumor development and drug resistance by creating an immunosuppressive microenvironment [26]. TAMs are highly heterogeneous and can exhibit different proinflammatory (M1-like) and anti-inflammatory (M2-like) phenotypes in different cancers and at different stages of the same cancer [26–28]. Some specific subsets of TAMs are associated with oncogenesis, angiogenesis, immunosuppression, and tumor metastasis [29–31], while TAMs can also mediate tumor phagocytosis and promote anti-tumor immunity [32–34]. The role of myeloid dendritic cells in tumors is currently understudied [35–37]. Th1 and Th2 are important immune cells in the vital TME, and the balanced regulation of Th1 and Th2 determines the outcome of the tumor immune response [37]. Th1 can secret anti-tumor cytokines, such as IFN-γ, TNF-α, and IL-2, which play an important role in anti-tumor immunity [37]. Low levels of Th1 are often associated with a poor prognosis [38–40]. Our study revealed that HTR7 expression was closely associated with the infiltration of monocytes, macrophages, myeloid dendritic cells, and Th1, which are closely linked to tumorigenesis, metastasis, and immunosuppression. Tumor immunotherapy targeting monocytes, macrophages, and Th1 cells has made significant progress [23,37]. Targeting specific immune cells may be therapeutic for HTR7-expressing tumors. Regarding immune checkpoints, our findings associate HTR7 with immune checkpoint pathways (e.g., PD-1/PD-L1, CTLA-4), suggesting that the clinical implementation of HTR7 testing could enhance patient stratification for immunotherapies.

DNA methylation is a critical component of gene regulation, playing a significant role in tumor development while also potentially being associated with TME. Hypermethylation of tumor suppressor genes facilitates uncontrolled cell growth and synergizes with oncogenic mutations to perpetuate cancer phenotypes. We found that the promoter methylation level of the HTR7 was increased in BRCA, COAD, PRAD, and STAD, corroborating the fact that HTR7 can play an oncogenic role in these cancers. Several recent studies have demonstrated that DNA methylation can remodel TME by regulating immune cell differentiation and infiltration patterns within tumors [41]. Our study suggested that aberrant methylation of HTR7 was associated with the infiltration of various immune cells, such as Th1, Th17, DCs, and macrophages. Several studies have shown that modulation of DNA methylation with immunotherapy may produce synergistic anti-tumor effects [41]. However, the relationship between gene expression, DNA methylation, and TME has not been adequately investigated, and it is hoped that more studies will reveal the relationship.

In depth, we explored cancer pathways and enrichment pathways associated with HTR7. The results showed that HTR7 could inhibit the cell cycle, DNA damage, and hormone AR pathways, while activating the EMT and RAS/MAPK pathways. GO and KEGG enrichment analyses revealed that HTR7 could participate in the G protein-coupled receptor signaling pathway, serotonin receptor signaling pathway, hormone signaling, cAMP signaling pathway, etc. The in vitro study by Jaya Gautam et al. on TNBC suggests that HTR7 is associated with the activation of PI3K/Akt and Ras/Raf/MAPK during the development of TNBC [11]. In vivo combined in vitro study by Xiaoli Sheng et al. also showed that HTR7 promotes laryngeal cancer proliferation and growth by activating the PI3K/AKT pathway [10]. These results are consistent with our findings. However, clinical and basic studies on the cancer pathway of HTR7 in most tumors are still scarce, and our results may inform further studies in the future.

Currently, numerous observational and basic/translational studies have confirmed the effectiveness of HTR7 in diagnosing tumors, predicting invasion or metastasis, and assessing prognosis in human tumor tissues [8,9,20,42–44]. Therapies targeting HTR7 are being investigated in several cancers. HTR7 receptor antagonists, such as SB-269970 and metergoline, have been shown to inhibit the proliferation of prostate, gastric, liver, and breast cancers in vitro or in vivo experiments [7,8,11,44,45]. Moreover, we mined the GDSC for drugs that may be effective against HTR7 expression, such as 5-fluorouracil, gemcitabine, sunitinib, tipifarnib, and trametinib. Existing studies suggest that targeting HTR7 has some potential in tumor therapy. Our results will be helpful for future HTR7-related clinical trials and drug screening.

There are some limitations of our study. Firstly, our study relied on retrospective bioinformatics analyses and lacked experimental validation of the biological role and expected drug sensitivity of HTR7. We aim to validate HTR7 as a predictive biomarker and explore potential therapeutic agents through future clinical studies. Secondly, we have employed extensive publicly accessible databases (e.g., TCGA, GTEx, TIMER). On the one hand, the heterogeneity of results may be due to differences in sample sources and assays. On the other hand, the generalizability of our findings may be compromised by the intrinsic constraints of these databases, including insufficient clinical follow-up and other sample biases.

## Conclusions

Our study suggested that HTR7 may have both pro-cancer and anti-carcinogenic effects, with specific diagnostic values and the ability to predict cancer prognosis. The expression of HTR7 is closely associated with TME, immune checkpoints, and DNA methylation. Our findings significantly enhance the understanding of HTR7’s role in cancer; nonetheless, further mechanistic investigations are necessary to reveal fundamentally unique pathways that may explain the dual functionality of HTR7 in cancer.

## Supporting information

S1 Fig The univariate regression and Kaplan−Meier curves for DSS in pan-cancer. High HTR7 expression was associated with unfavorable DSS in BLCA, ESCA, GBM, HNSC, PAAD, STAD, THYM, and UVM. High HTR7 expression was associated with favorable DSS in ACC, KIRC, KIRP, LUSC, PRAD, READ, and SKCM. The Kaplan-Meier method and Cox regression were used.
(TIF)

S2 Fig The univariate regression and Kaplan−Meier curves for PFI in pan-cancer. High HTR7 expression was associated with unfavorable PFI in BLCA, BRCA, GBM, HNSC, PAAD, STAD, THYM, and UVM. High HTR7 expression was associated with favorable PFI in ACC, CESC, COAD, KIRC, KIRP, and UCS. The Kaplan-Meier method and Cox regression were used.
(TIF)

S3 Fig The univariate regression and Kaplan−Meier curves for DFI in pan-cancer. High HTR7 expression was associated with unfavorable DFI in BLCA, DLBC, and ESCA. High HTR7 expression was associated with favorable DFI in ACC, CESC, COAD, LUSC, PRAD, TGCT, UCS, and UVM. The Kaplan-Meier method and Cox regression were used.
(TIF)

S4 Fig Chromosomal distribution of the methylation probes associated with HTR7. HTR7 had 11 methylation probes, including cg10650018, cg05756489, cg06291867, cg15364725, cg01274715, cg00967901, cg26135506, cg26332534, cg12583095, cg23720528 and cg12639324.
(TIF)

S5 Fig Co-expressed genes of HTR7. Twenty genes were closely related to HTR7, involving multiple networks such as physical interactions, co-expression, and co-localization.
(TIF)

## Abbreviations

ACC: Adrenocortical carcinoma
BLCA: Bladder Urothelial Carcinoma
BRCA: Breast invasive carcinoma
CESC: Cervical squamous cell carcinoma and endocervical adenocarcinoma
CHOL: Cholangiocarcinoma
COAD: Colon adenocarcinoma
DLBC: Lymphoid Neoplasm Diffuse Large B-cell Lymphoma
ESCA: Esophageal carcinoma
GBM: Glioblastoma multiforme
HNSC: Head and Neck squamous cell carcinoma
KICH: Kidney Chromophobe
KIRC: Kidney renal clear cell carcinoma
KIRP: Kidney renal papillary cell carcinoma
LAML: Acute Myeloid Leukemia
LGG: Brain Lower Grade Glioma
LIHC: Liver hepatocellular carcinoma
LUAD: Lung adenocarcinoma
LUSC: Lung squamous cell carcinoma
MESO: Mesothelioma
OV: Ovarian serous cystadenocarcinoma
PAAD: Pancreatic adenocarcinoma
PCPG: Pheochromocytoma and Paraganglioma
PRAD: Prostate adenocarcinoma
READ: Rectum adenocarcinoma
SARC: Sarcoma
SKCM: Skin Cutaneous Melanoma
STAD: Stomach adenocarcinoma
TGCT: Testicular Germ Cell Tumors
THCA: Thyroid carcinoma
THYM: Thymoma
UCEC: Uterine Corpus Endometrial Carcinoma
UCS: Uterine Carcinosarcoma
UVM: Uveal Melanoma.
