# Supplementary figures and images for: Comprehensive bioinformatic analysis of HTR7: A potential biomarker for diagnosis, survival, and immunotherapy in pan-cancer
Source: PLoS One. 2025 Nov 14;20(11):e0335398. doi: 10.1371/journal.pone.0335398 (PMC12617886; doi:10.1371/journal.pone.0335398)

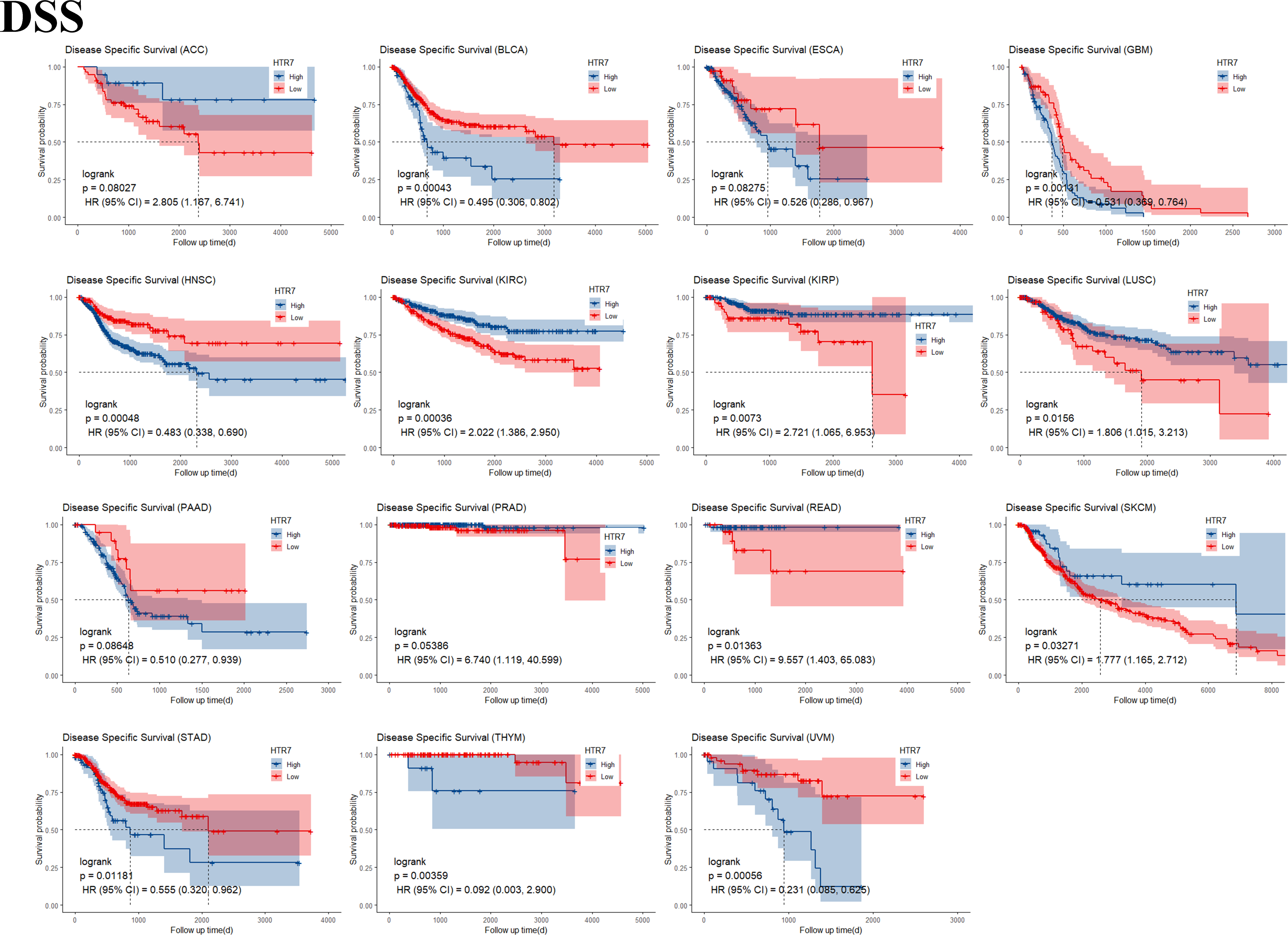

Supplement: S1 Fig — High HTR7 expression was associated with unfavorable DSS in BLCA, ESCA, GBM, HNSC, PAAD, STAD, THYM, and UVM. High HTR7 expression was associated with favorable DSS in ACC, KIRC, KIRP, LUSC, PRAD, READ, and SKCM. The Kaplan-Meier method and Cox regression were used. (TIF) [file pone.0335398.s001.tif]

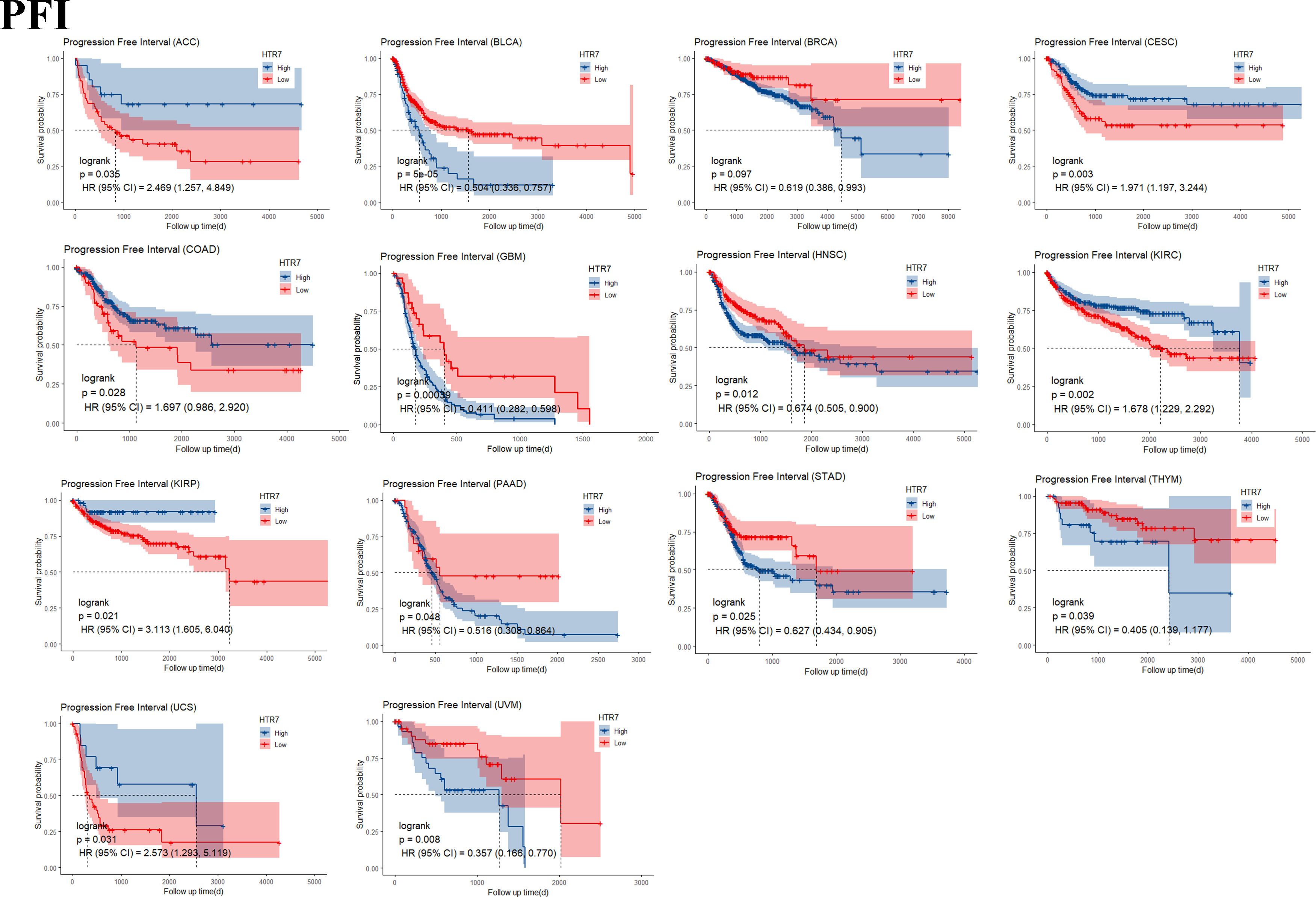

Supplement: S2 Fig — High HTR7 expression was associated with unfavorable PFI in BLCA, BRCA, GBM, HNSC, PAAD, STAD, THYM, and UVM. High HTR7 expression was associated with favorable PFI in ACC, CESC, COAD, KIRC, KIRP, and UCS. The Kaplan-Meier method and Cox regression were used. (TIF) [file pone.0335398.s002.tif]

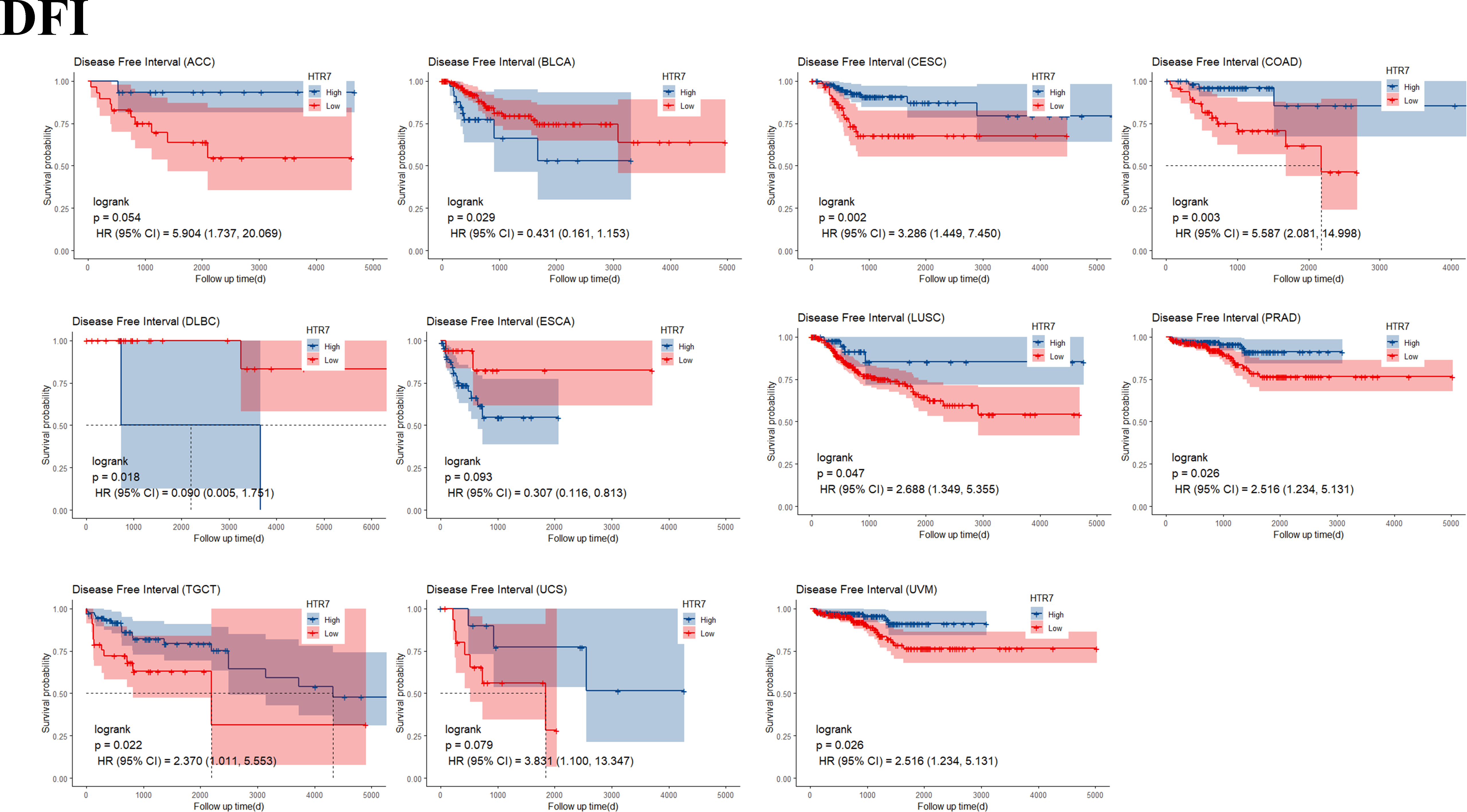

Supplement: S3 Fig — High HTR7 expression was associated with unfavorable DFI in BLCA, DLBC, and ESCA. High HTR7 expression was associated with favorable DFI in ACC, CESC, COAD, LUSC, PRAD, TGCT, UCS, and UVM. The Kaplan-Meier method and Cox regression were used. (TIF) [file pone.0335398.s003.tif]

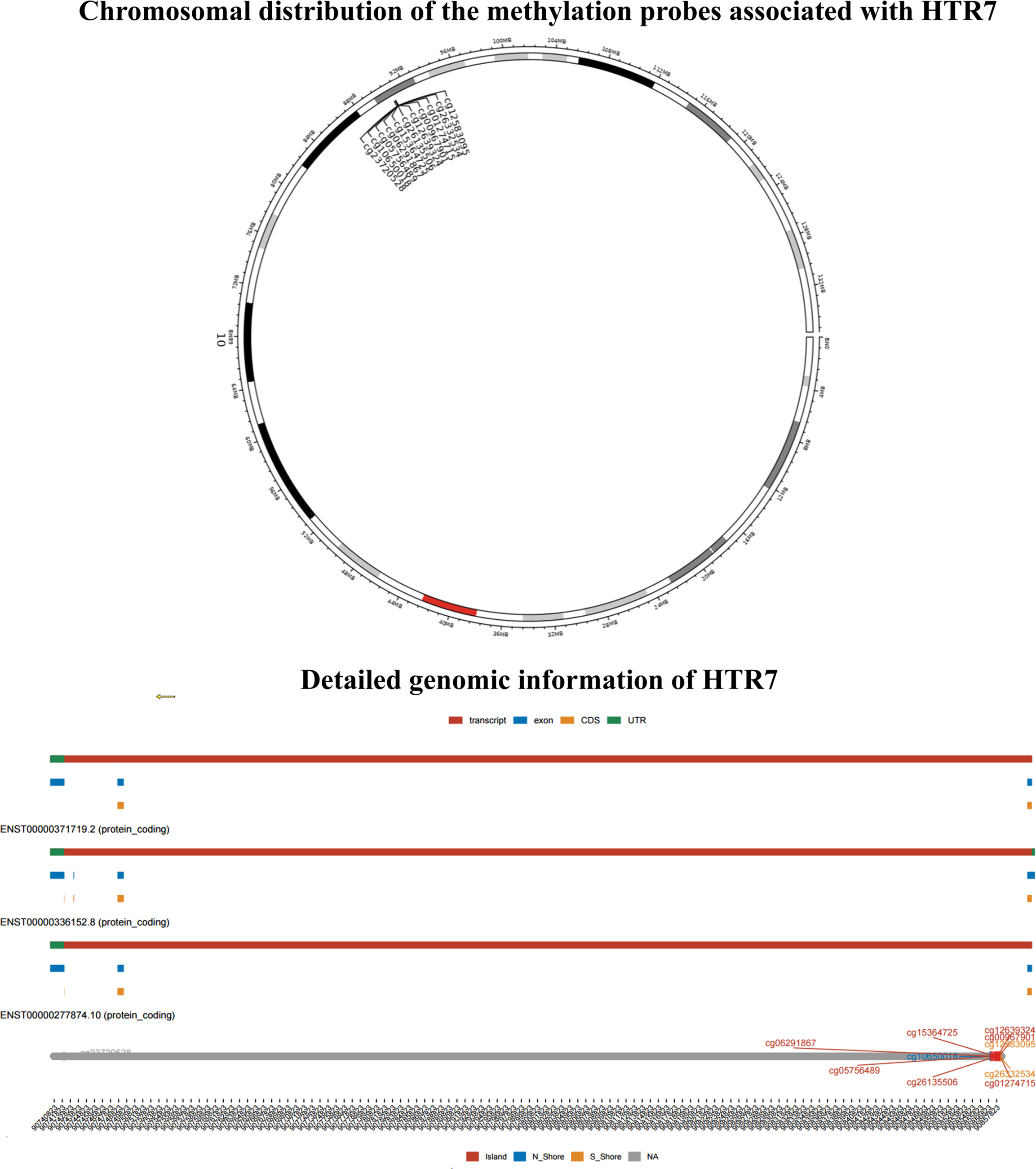

Supplement: S4 Fig — HTR7 had 11 methylation probes, including cg10650018, cg05756489, cg06291867, cg15364725, cg01274715, cg00967901, cg26135506, cg26332534, cg12583095, cg23720528 and cg12639324. (TIF) [file pone.0335398.s004.tif]

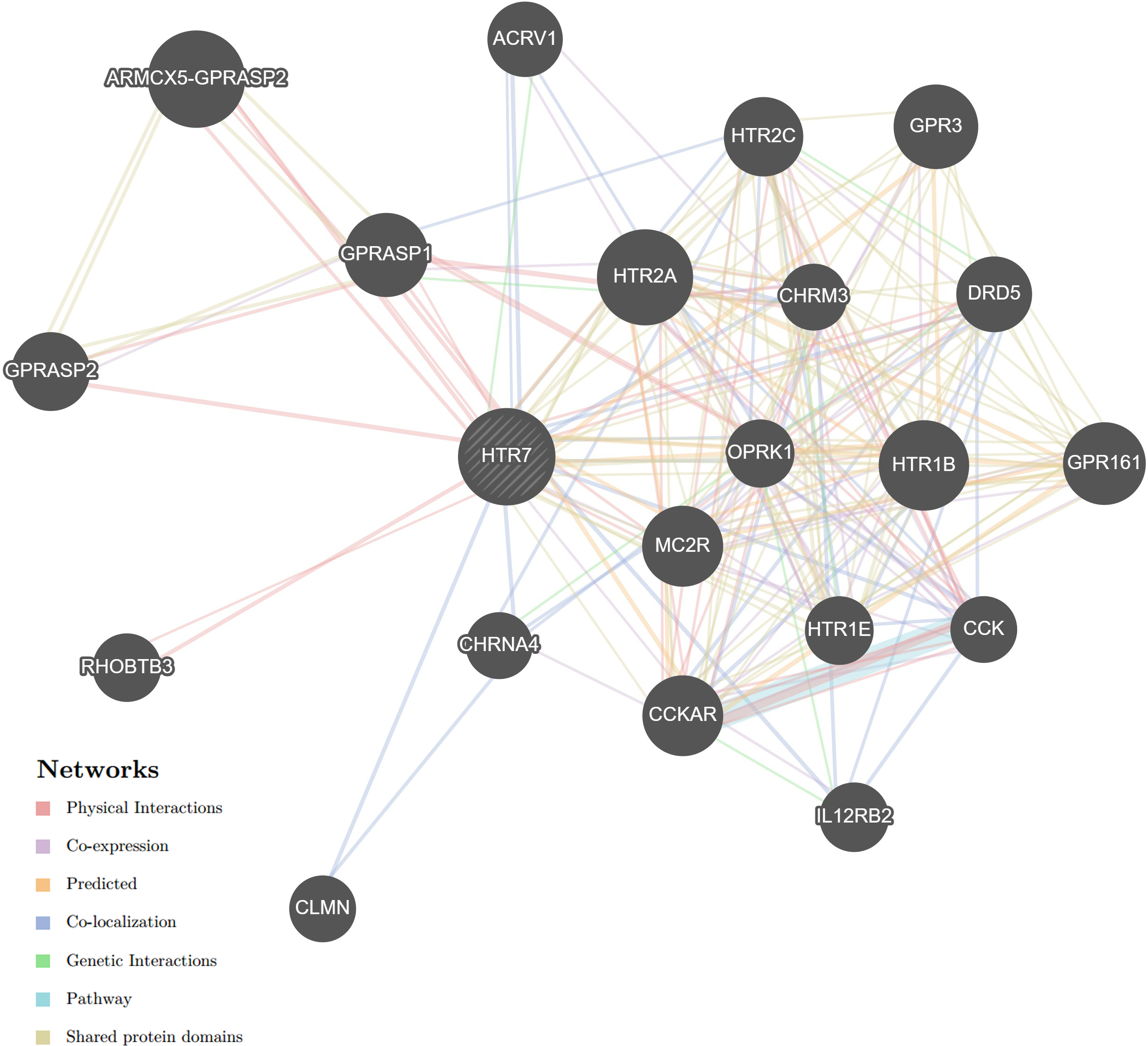

Supplement: S5 Fig — Twenty genes were closely related to HTR7, involving multiple networks such as physical interactions, co-expression, and co-localization. (TIF) [file pone.0335398.s005.tif]
